# Transthoracic echocardiography for imaging of the different coronary artery segments: a feasibility study

**DOI:** 10.1186/1476-7120-7-58

**Published:** 2009-12-22

**Authors:** Johnny Vegsundvåg, Espen Holte, Rune Wiseth, Knut Hegbom, Torstein Hole

**Affiliations:** 1 Department of Internal Medicine, Ålesund Hospital, Ålesund, Norway; 2 Department of Circulation and Medical Imaging, Norwegian University of Science and Technology (NTNU), Trondheim, Norway; 3 Department of Cardiology, Trondheim University Hospital, Trondheim, Norway; 4 Medical Faculty, Norwegian University of Science and Technology (NTNU), Trondheim, Norway

## Abstract

**Background:**

Transthoracic echocardiography (TTE) may be used for direct inspection of various parts of the main coronary arteries for detection of coronary stenoses and occlusions. We aimed to assess the feasibility of TTE to visualise the complete segments of the left main (LM), left descending (LAD), circumflex (Cx) and right (RCA) coronary arteries.

**Methods:**

One hundred and eleven patients scheduled for diagnostic coronary angiography because of chest pain or acute coronary syndrome had a TTE study to map the passage of the main coronary arteries. LAD, Cx and RCA were each divided into proximal, middle and distal segments. If any part of the individual segment of a coronary artery with antegrade blood flow was not visualised, the segment was labeled as not satisfactorily seen.

**Results:**

Complete imaging of the LM was achieved in 98% of the patients. With antegrade directed coronary artery flow, the proximal, middle and distal segments of LAD were completely seen in 96%, 95% and 91% of patients, respectively. Adding the completely seen segments with antegrade coronary flow and segments with retrograde coronary flow, the proximal, middle and distal segments of LAD were adequately visualised in 96%, 96% and 93% of patients, respectively. With antegrade directed coronary artery flow, the proximal, middle and distal segments of Cx were completely seen in 88%, 61% and 3% and in RCA in 40%, 28% and 54% of patients. Retrograde coronary artery flow was correctly identified as verified by coronary angiography in seven coronary segments, mainly in the posterior descending artery (labeled as the distal segment of RCA) and distal LAD.

**Conclusions:**

TTE is a feasible method for complete demonstration of coronary flow in the LM, the proximal Cx and the different segments of LAD, but less suitable for the RCA and mid and distal segments of the Cx. (ClinicalTrials.gov number NTC00281346.)

## Background

Non-invasive imaging of coronary arteries by transthoracic echocardiography (TTE) is an emerging diagnostic tool for studying flow in the left main (LM), the left descending artery (LAD), the circumflex (Cx) and the right coronary arteries (RCA) [1,2]. Direct visualisation of segments of the coronary arteries may help in diagnosing significant coronary artery stenoses [2-4]. With this technique, a coronary stenosis typically exhibits local flow acceleration and turbulence expressed as colour aliasing by colour flow Doppler and accelerated flow velocities across the stenosis [2-5]. Total occlusion of a coronary artery may be detected by retrograde flow in the same artery [6-9]. However, demonstration of stenosis or retrograde flow in the main coronary arteries by TTE is dependent on optimal visualisation of the different segments of each main coronary artery. The aim of this study was to assess the feasibility of TTE to visualise the complete segments of the LM, LAD, Cx and RCA in a larger set of patients.

## Methods

### Study population

Patients were consecutively included in the study if they fulfilled the following criteria: (i) already scheduled for diagnostic coronary angiography because of chest pain (typical or atypical angina pectoris), or coronary angiography was planned because of acute coronary syndrome; (ii) patient age above 18 years; (iii) met no exclusion criteria. The exclusion criteria were: (i) previous coronary artery bypass surgery; (ii) presumed insufficient acoustic windows because of severe emphysema or gross overweight; (iii) significant valvular disease; (iv) atrial fibrillation; (v) administrative reasons (logistics).

The study protocol was approved by the Regional Committee for Medical and Health Research Ethics and the Norwegian Data Inspectorate. All participants gave written, informed consent. ClinicalTrials.gov number NTC00281346.

Six patients did not enter the study because of insufficient acoustic windows (n = 3), lack of consent (n = 2) or aortic stenosis (n = 1). We included 115 patients in the study, but 4 patients were later excluded from further analysis because of protocol violation: aortic stenosis (n = 2), atrial fibrillation (n = 2). The final study group consisted of 111 patients. Clinical characteristics of the patients are presented in Table 1. All patients took their prescribed medication the day of the echocardiographic study (Table 1).

**Table 1:** Baseline characteristics of the study cohort (n = 111)

	No of subjects (%) mean ± SD
Age (years)	62,9 ± 9,6

Heart rate (strokes/minute)	63 ± 7,4

BMI (kg/m^2^)	26 ± 3,6

Male sex	82 (74,0)

Total cholesterol (mmol/L)	4,9 ± 1,1

Blood pressure (mm Hg)	

Systolic	141 ± 20

Diastolic	82 ± 12

Medical history	

Hypertension (>140/90 mm Hg)	61 (55,0)

Current smoking	29 (26,1)

Diabetes	11 (9,9)

Previous CAD	24 (21,6)

ACS	35 (31,5)

Cardiac medication	

Aspirin	98 (88,3)

Thienopyridine	38 (34,2)

Low-molecular-weight heparin	30 (27,0)

β-Blockers	87 (78,4)

Statins	89 (80,2)

Calcium antagonists	21 (18,9)

ACE-inhibitors/ARB	25 (22,5)

Organic nitrate, daily maintenance	13 (11,7)

BMI = body mass index, CAD = coronary artery disease, ACS = acute coronary syndrome, ACE = angiotensin-converting enzyme, ARB = angiontensin II receptor blocker

### Echocardiographic evaluation

Patients were examined with an Acuson Sequoia c 512 (Siemens Medical Solutions Inc, USA) ultrasound system connected to standard 4V1C and 7V3C transthoracic transducers. The anatomical course of the coronary arteries was examined by use of colour Doppler mapping with data postprocessing mix function, which makes the colours transparent. The velocity scale of colour Doppler was set to 0,24 m/s, but was actively changed to provide optimal images. Contrast agent was not used.

**Figure 1:**
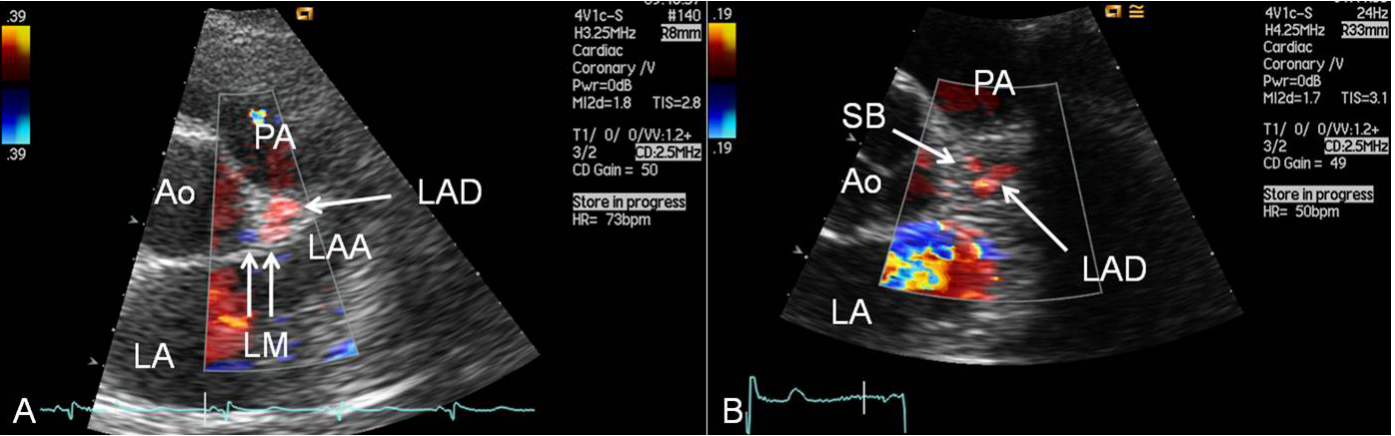
**Examples of antegrade coronary artery flow in the LM and proximal parts of LAD**. (A) In modified parasternal short-axis view the left main coronary artery (LM) is seen leaving the left sinus of Valsalva and continuing as the left anterior descending coronary artery (LAD) turning slightly towards the transducer. (B) In modified parasternal short-axis view the first septal branch (SB) is seen leaving the LAD. Ao = aortic root; LA = left atrium; LAA = left atrial appendage; PA = pulmonary artery.

**Figure 2:**
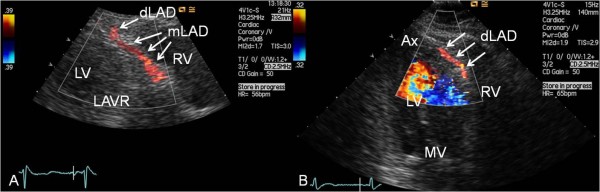
**Examples of antegrade coronary artery flow in the middle and distal segments of LAD**. (A) The middle left anterior descending coronary artery (mLAD) is imaged from parasternal modified long-axis view focusing on the anterior interventricular sulcus. (B) The distal left anterior descending coronary artery (dLAD) is seen from modified apical 3-chamber view focusing on the anterior interventricular sulcus. Ax = apex of the left ventricle; LAVR = left atrioventricular ring; LV = left ventricle; MV = mitral valve; RV = right ventricle.

**Figure 3:**
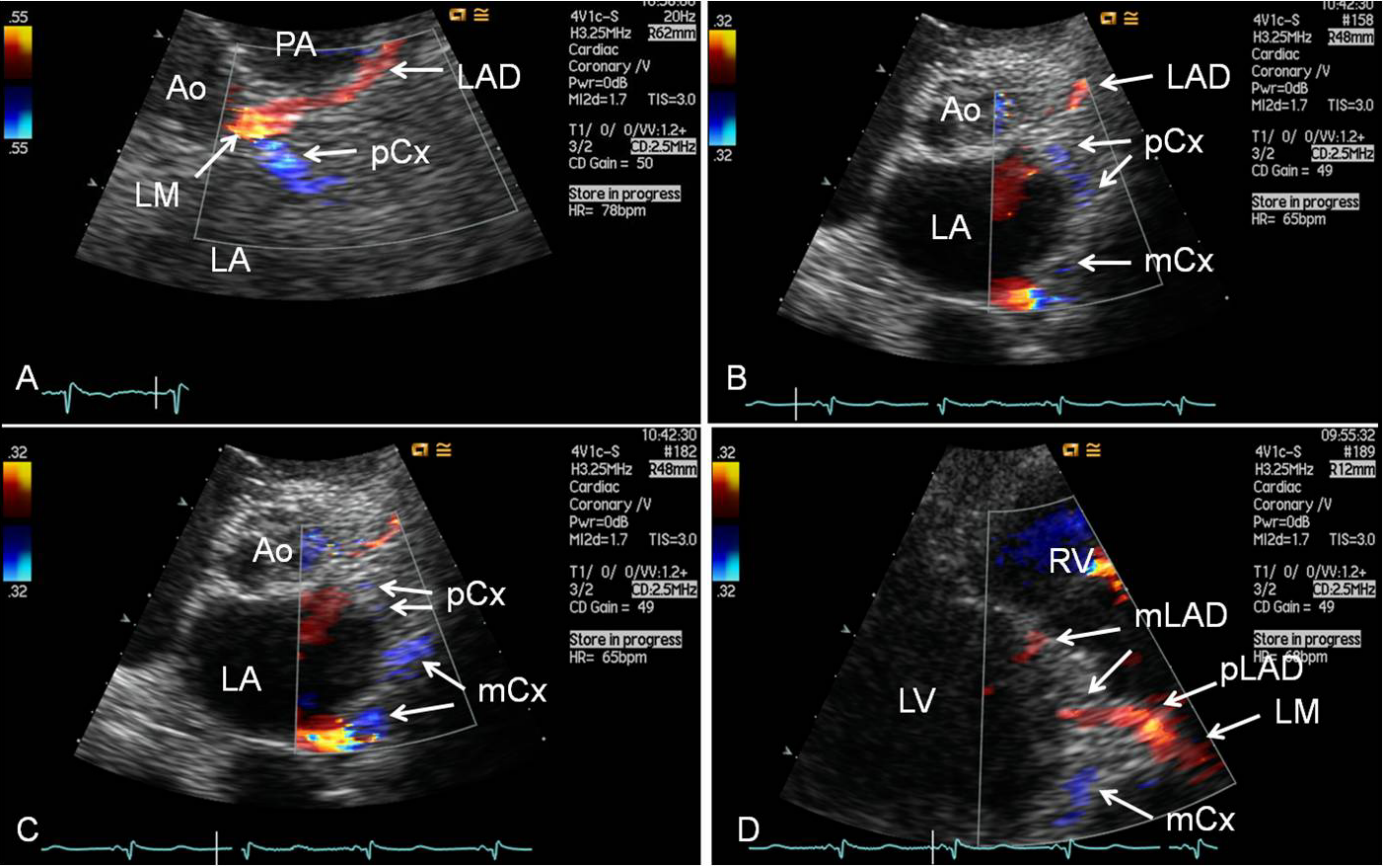
**Examples of antegrade coronary artery flow in the proximal and middle segments of Cx**. (A) In modified parasternal short-axis view focusing on the area adjacent to the left sinus of Valsalva the proximal circumflex coronary artery (pCx) is seen leaving the left main coronary artery (LM). (B and C) In modified parasternal short-axis views the proximal and middle segments of the circumflex coronary artery (Cx) are found passing caudally in the lateral atrioventricular sulcus. (D) From parasternal modified long-axis view focusing on the lateral atrioventricular sulcus the middle Cx (mCx) is seen traversing caudally. Ao = aortic root/valve; LA = left atrium; LAD = left anterior descending coronary artery; LV = left ventricle; mLAD = middle segment of LAD; PA = pulmonary artery; pLAD = proximal segment of LAD; RV = right ventricle.

**Figure 4:**
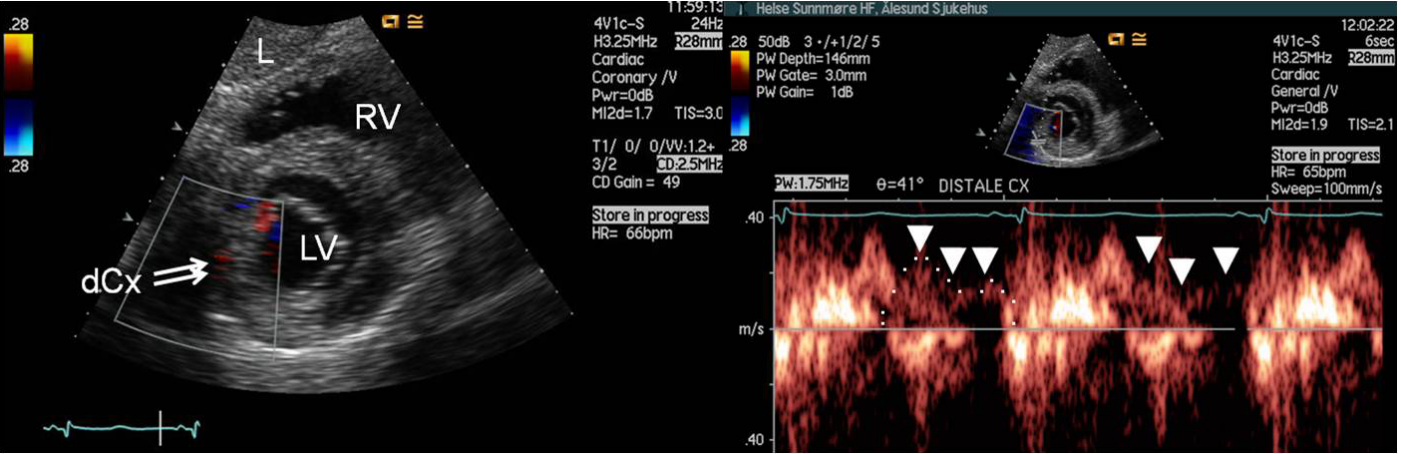
**Example of antegrade coronary artery flow in the distal segment of Cx**. The distal segment of the circumflex coronary artery (dCx) imaged by colour Doppler mapping and matching spectral Doppler tracings of blood flow (with arrowheads denoting diastolic flow waveform with one diastolic flow velocity waveform enveloped), imaged from modified subcostal short-axis view focusing on the inferior atrioventricular sulcus. L = liver; LV = left ventricle; RV = right ventricle.

**Figure 5:**
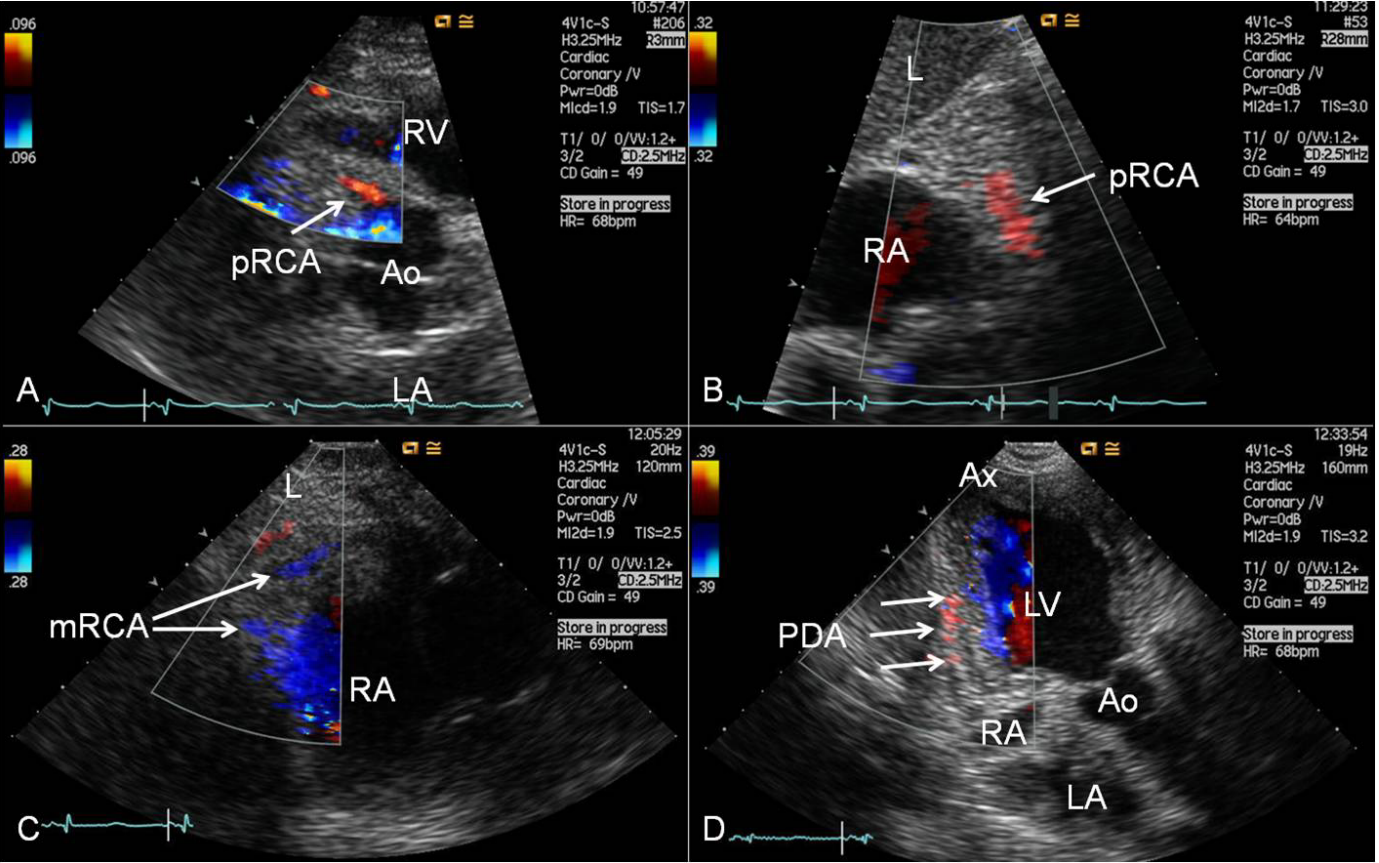
**Examples of antegrade coronary artery flow in the RCA and PDA**. (A) In modified parasternal short-axis view searching the area adjacent to the right sinus of Valsalva the proximal right coronary artery (pRCA) is seen leaving the aortic root (Ao). (B) From subcostal modified sagittal view the pRCA is seen traversing caudally on the anterior tricuspid ring. (C) Using subcostal modified short-axis view parts of the middle segment of the right coronary artery (mRCA) are seen coursing medially on the medial tricuspid ring. (D) From modified apical 2-chamber view focusing on the posterior interventricular sulcus parts of the posterior descending coronary artery (PDA) are seen coursing toward the apex (Ax). L = liver; LA = left atrium; LV = left ventricle; RA = right atrium; RV = right ventricle.

With the patient in the supine, left and right lateral decubitus positions, all standard and modified apical, parasternal, and subcostal views were used to follow the course of the LM, LAD, Cx and RCA, from the start of each artery and distally as far as possible. With the patient in the left lateral decubitus position, the LM was examined from the left parasternal short- and long-axis views focusing on the area adjacent to the left sinus of Valsalva cranial to the aortic valve (Figure 1A). In the same short- and long-axis views the proximal LAD (pLAD) could be seen leaving the LM and turning slightly towards the transducer (Figure 1A). The LM and pLAD could also be imaged from modified apical 5- and 2-chamber views in many patients. Origin of the first septal branch was often identified, marking the transition from the pLAD to the middle segment of LAD (mLAD) (Figure 1B). If the first septal branch was not visualised, the division between the pLAD and mLAD was set approximately halfway to the level of the left ventricular papillary muscles. The course of the mLAD and distal LAD (dLAD) was imaged from parasternal modified short- and long-axis views focusing on the anterior interventricular sulcus, and the same arterial segments could also be seen from modified apical 2- and 3-chamber views (Figure 2). The level of the left ventricular papillary muscles marked the division between the mid and distal segments of the LAD. The origin and proximal part of the Cx (pCx) was found by using the same views as searching for the LM. By focusing on the atrioventricular sulcus, the pCx was seen passing in front of the left atrial appendage. The level of the inferor wall of the left atrial appendage marked the division between the pCx and middle segment of the Cx (mCx). By modified parasternal short-axis views, and sometimes subcostal short-axis views, mCx was visualised as it passed caudally in the atrioventricular sulcus to the inferior margin of the sulcus, and the artery continued further in the atrioventricular sulcus as the distal segment of the Cx (dCx). The different segments of Cx are imaged in Figure 3 and 4. With the patient in the right or left decubitus position using parasternal short-axis views, the proximal segment of the RCA (pRCA) was looked for in the area adjacent to the right sinus of Valsalva cranial to the aortic valve (Figure 5A). The rest of the pRCA was visualised from supine subcostal modified sagittal and 4-chamber views focusing on the anterior tricuspid ring (Figure 5B). The pRCA was considered as the part of RCA passing anterior surface of the tricuspid ring as far as the inferior margin of the right ventricle. Since we had some problems defining the transition between the middle and distal part of the RCA, these segments were labeled together as the middle segment of RCA (mRCA). This combined arterial segment was seen by focusing on the medial and posterior tricuspid ring using subcostal modified short-axis and 4-chamber views (Figure 5C). We labeled the posterior descending artery (PDA) as the distal segment of RCA, and this segment was imaged from modified apical 2-chamber views coursing toward the apex in the posterior interventricular sulcus (Figure 5D).

**Figure 6:**
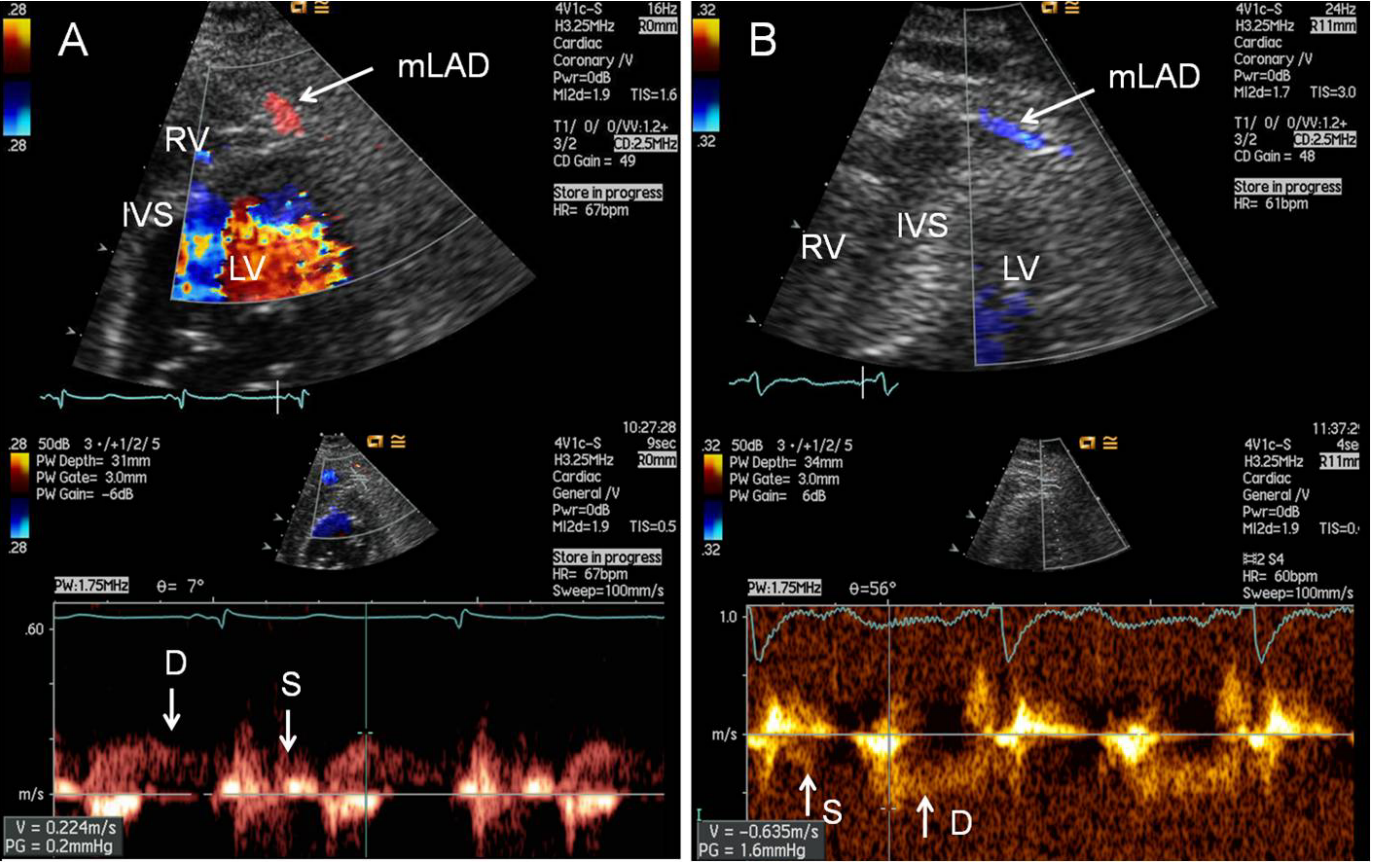
**Examples of antegrade and retrograde flow in the middle segment of LAD**. The middle segment of the left anterior descending coronary artery (mLAD) imaged by colour Doppler mapping and matching spectral Doppler tracings of blood flow, imaged from modified parasternal short-axis view focusing on the anterior interventricular sulcus: (A) The mLAD is seen with antegrade flow. (B) The mLAD is seen with retrograde flow. D = spectral Doppler tracings of diastolic coronary blood flow; IVS = interventricular septum; LV = left ventricle; RV = right ventricle; S = spectral Doppler tracings of systolic coronary blood flow.

The coronary artery flow velocity waveform appears as a complex of a small wave in systole and a large trapezoid wave in diastole (Figure 6A). The coronary artery antegrade flow velocities could be low, normal or elevated, and was not always measured. The colour Doppler flow could either be laminar or turbulent. When colour flow Doppler recordings indicated reversed coronary artery flow (the functional diagnosis of coronary obstruction more proximal in the coronary artery), we distinguished the retrograde coronary artery flow from coronary venous flow by finding inverted coronary flow velocity waveform (Figure 6B). In contrast, the coronary venous flow appears as a prominent systolic flow wave.

As outlined above, the LM had one segment and the other main coronary arteries (LAD, Cx and RCA) had each a proximal, middle and distal segment. For each segment three different possibilities were defined: (i) the segment was completely visualised; (ii) the segment was not satisfactorily visualised if any part of the individual segment was not seen or the segment was not visualised at all; (iii) the segment was defined with retrograde flow.

### Coronary angiography

Coronary angiography was performed using standard techniques. All angiograms were classified according to left or right dominance. Collateral flow to occluded arteries was graded according to the Rentrop classification (grade 0, no visible filling of any collateral channel, grade 1, filling of side branches of the occluded artery, grade 2, partial filling of the epicardial vessel and grade 3, complete collateral filling of the epicardial vessel) [10].

### Statistical analysis

Continuous variables are presented as mean and standard deviation (SD), or median (5-95 percentiles) if the variables were skewed. Categorical variables are presented as fractions/percent. Univariate regression analyses were used to explore relationships between success rate and demographic and clinical variables. Variables in univariate testing with p < 1.10 were entered in multivariate forward analyses. The success rates among different patient sub-populations were compared using the unconditional z-pooled test for binomial proportions, with confidence interval method with coefficient 0.9999. P < 0.05 was considered statistically significant. All analyses were done with SPSS for windows (SPSS Inc, Chicago, IL, v 15,0).

## Results

### Visualisation of different coronary artery segments

#### Left main coronary artery

Complete imaging of the LM was achieved in all but two patients (98%) (Table 2).

**Table 2:** Findings of visualisation of individual coronary artery segments

Segment	Antegrade flow or retrograde flow	Antegrade flow	Retrograde flow
	n (%)	n (%)	n (%)
LM	109 (98,2)	109 (98,2)	0 (0)
pLAD	106 (95,5)	106 (95,5)	0 (0)
mLAD	106 (95,5)	105 (94,6)	1 (0,9)
dLAD	103 (92,8)	101 (91,0)	2 (1,8)
pCx	98 (88,3)	98 (88,3)	0 (0)
mCx	68 (61,3)	68 (61,3)	0 (0)
dCx	3 (2,7)	3 (2,7)	0 (0)
pRCA	44 (39,6)	44 (39,6)	0 (0)
mRCA	32 (28,8)	31 (27,9)	1 (0,9)
PDA	65 (58,6)	60 (54,1)	5 (4,5)

Findings of complete visualisation of coronary artery segments with antegrade flow or coronary segments found with retrograde flow (n = 111). LM = left main coronary artery, pLAD = proximal segment of left anterior descending artery, mLAD = middle segment of left anterior descending artery, dLAD = distal segment of left anterior descending artery, pCx = proximal segment of circumflex coronary artery, mCx = middle segment of circumflex coronary artery, dCx = distal segment of circumflex coronary artery, pRCA = proximal segment of right coronary artery, mRCA = middle segment of right coronary artery, PDA = posterior descending artery

#### Left anterior descending artery

Adding the patients with completely seen segments with antegrade coronary flow and patients with segmental retrograde coronary flow, the proximal, middle and distal segments of LAD were adequately visualised in 96%, 96% and 93% of the patients, respectively (Table 2). With antegrade directed coronary artery flow, the proximal, middle and distal segments of LAD were entirely seen in 96%, 95% and 91% of the patients, respectively (Table 2). Retrograde coronary artery flow was seen in one middle and two distal segments of the LAD (Table 2). Coronary angiography demonstrated total occlusion of LAD in these cases with collateral flow to the occluded artery in Rentrop class ≥ 2. Visualisation of both LM and pLAD or LM and pLAD/mLAD was achieved in 95% and 90% of the patients (Table 3). Visualisation of the LM together with all segments of LAD was achieved in 85% of patients (Table 3). The visualisation of LM and the complete LAD was negatively related to patients' body mass index (BMI) (p = 0.002) in the regression analyses.

**Table 3:** Visualisation of several coronary artery segments combined

	No of subjects (%)
LM + pLAD + mLAD + dLAD	94 (84,7)
LM + pLAD	105 (94,6)
LM + pLAD + mLAD	100 (90,1)
pCx + mCx + dCx	3 (2,7)
pCx + mCX	68 (61,3)
pRCA + mRCA + PDA	17 (15,3)
pRCA + PDA	30 (27,0)
LM + all segments of LAD/CX/RCA	1 (0,9)
LM + pLAD + mLAD + pCx + mCx + pRCA + mRCA	14 (12,6)
LM + pLAD + pCx + pRCA	39 (35,1)
LM + pLAD + pCx	94 (84,7)

Visualisation of several coronary artery segments combined, with each individual coronary segment either completely imaged with antegrade flow or found with retrograde flow (n = 111). LM = left main coronary artery, pLAD = proximal segment of left anterior descending artery, mLAD = middle segment of left anterior descending artery, dLAD = distal segment of left anterior descending artery, pCx = proximal segment of circumflex coronary artery, mCx = middle segment of circumflex coronary artery, dCx = distal segment of circumflex coronary artery, pRCA = proximal segment of right coronary artery, mRCA = middle segment or right coronary artery, PDA = posterior descending artery

#### Circumflex coronary artery

With antegrade directed coronary artery flow, the proximal, middle and distal segments of Cx were completely seen in 88%, 61% and 3% of the patients, respectively (Table 2). No patient was found to have retrograde flow. Both pCx and mCx could be visualised in 61% of patients, while all three segments of Cx could be seen in 3% of patients (Table 3). Visualisation of the complete Cx or the proximal and mid segments was negatively related to patient age (p = 0,03) in univariate regression analyses. Visualisation of the complete Cx was negatively related to age (p = 0,01) and heart rate (p = 0,03) in multivariate analyses.

#### Right coronary artery

Adding the patients with completely seen segments with antegrade coronary flow and patients with segmental retrograde coronary flow, the pRCA, mRCA and PDA were adequately visualised in 40%, 29% and 59%, respectively (Table 2). With antegrade directed coronary artery flow, the proximal and middle segments of the RCA and PDA were entirely seen in 40%, 28% and 54%, respectively (Table 2). Retrograde coronary artery flow was seen in one mRCA and five PDAs (Table 2). Coronary angiography showed corresponding findings apart from two PDAs which had antegrade flow on angiography. In the cases where retrograde Doppler flow in PDA was confirmed angiographically collateral circulation in PDA was Rentrop class ≥ 2. Both the pRCA and PDA were visualised in 27% of patients, while the total RCA/PDA was adequately imaged in 15% of patients (Table 3). Visualisation of the complete RCA/PDA was negatively related to patient BMI in regression analyses (p = 0.01).

### Visualisation of proximal versus distal coronary segments

Visualisation of all segments of the coronary arteries was achieved in only one patient (1%) (Table 3). There were significant differences in the visualisation of proximal versus distal segments between the different coronary arteries. In contrast with Cx and RCA, the rate of visualisation of distal and proximal segments of LAD was equal (Table 2). In Cx the distal segment was visualised in only a minority of patients. In RCA it was the middle segment which was most difficult to visualise (Table 2). The LM and proximal segments of LAD, Cx and RCA were visualised in 35% of patients. Excluding RCA, LM and proximal segments of LAD and Cx were adequately seen in 85% of patients (Table 3).

### Right versus left coronary dominance

Coronary angiograms were available for 108 patients, and 8 patients were found to have dominant Cx, supplying the PDA from the distal continuation of the Cx. The remaining patients had the RCA as the dominant artery, supplying the PDA. Among the eight patients with left coronary dominance, the pCx, mCx and dCx were completely visualised in seven, six and one patients, respectively. Among the same patients, the pRCA, mRCA and PDA were completely visualised in zero, one and two patients, respectively. The statistical analyses showed a trend towards lower feasibility of adequate visualisation of pRCA and mRCA and a trend towards higher feasibility of visualisation of pCx and mCx when the Cx was the dominant coronary artery.

### Visualisation of different coronary artery segments according to clinical presentation

Statistical analyses showed no significant differences in the degree of adequate visualisation of the various main coronary segments when comparing patients with or without acute coronary syndrome, or comparing patients with or without known coronary disease.

## Discussion

In this study of consecutively included patients with suspected or definite coronary artery disease the feasibility of transthoracic echocardiography for visualisation of different segments of the main coronary arteries was examined. The purpose of the study was to elucidate the feasibility of viewing the complete coronary artery segments with antegrade flow or findings of retrograde coronary artery flow. The main finding was that the left main, the LAD segments and the proximal Cx segment could be completely imaged in the great majority of patients while the mid and distal Cx segments and the RCA were less well imaged.

Use of TTE for direct inspection of various parts of the main coronary arteries has been the subject of several studies, both for detection of coronary stenoses and occlusions [2-9,11-15]. Coronary occlusions may be detected by retrograde flow in the coronary artery [6-9]. Coronary stenoses can be identified by local flow acceleration and turbulence expressed as colour aliasing by colour flow Doppler and accelerated flow velocities across the stenosis, with further quantification of the individual stenosis by comparing flow velocities at the site of aliasing with nearest upstream non-accelerated prestenotic flow velocities [2-5,11,12]. A stenotic to prestenotic flow velocity ratio (SPVR) exceeding 2 has been proposed as a cut-off value for significant stenosis [2,3]. Accelerated coronary artery flow velocities typically give a stronger colour Doppler signal than normal laminar coronary flow, and these turbulent flow signals may be detected without visualisation of adjacent up- or downstream coronary portions [2]. However, entire imaging of the individual coronary segment should ease the demonstration of possible stenoses with further Doppler measurements of flow velocities. So far, the largest study identifying stenoses by searching the various main coronary artery segments for flow turbulence and a stenotic SPVR included 84 patients, without performing detailed analyses on the visibility of the different coronary artery segments by TTE [2]. In contrast with previous studies, our study tried to elucidate the feasibility of complete visualisation of the various main coronary artery segments, in a larger set of patients with various degrees of atherosclerotic coronary disease.

Findings of retrograde flow in the main coronary arteries by TTE have high accuracy in documenting LAD occlusions [6-8] and RCA/PDA occlusions [7,9]. Retrograde coronary artery flow was rarely found in our patient cohort; in only nine main coronary artery segments, mainly in the PDA and dLAD (Table 2). Our findings of retrograde flow by TTE corresponded well with findings by coronary angiography, with matching findings in seven of nine patients. In the two patients falsely found with retrograde flow in the PDA by TTE, we suspect that the distal part of PDA was confused with the recurrent branch of the dLAD running around the apex of the heart.

Although large portions of any coronary artery segment with antegrade flow often could be satisfactorily seen in our study, the whole segment (with antegrade flow) had to be imaged to be labeled completely visualised, as outlined above. Complete visualisation of the various main coronary artery segments was dependent on both the artery and segment investigated (Table 2). Our study demonstrates that TTE is a feasible method for complete visualisation of different coronary artery segments with antegrade flow, especially the LM, the proximal segments of LAD and Cx, and the middle and distal segments of LAD. The distal Cx and the RCA segments were not adequately visualised. This may be due to several factors including individual variations in coronary anatomy for the Cx and the RCA. Even with use of invasive coronary angiography the distal segments of Cx may be difficult to define in some patients. The small study group with left coronary dominance showed a trend towards more complete visualisation of the proximal and mid segments of Cx and of less adequate visualisation of pRCA and mRCA. Patients' BMI was a significant predictor of success rate in our study. Our results are indicative of what can be expected with this technique in the hands of experienced echocardiographers and currently available equipment. Further developments of echocardiographic technology may both increase the feasibility of visualisation of coronary arteries by TTE and diminish echocardiographic limitations caused by overweight.

Earlier studies using coronary colour flow Doppler TTE are not directly comparable to our study, since those studies primarily focused on searching for coronary artery stenoses and occlusions [2-4,6-9,11-14], without fully describing the degree of completeness of imaging of the coronary artery segment investigated. Several of these studies only concentrated on the LM and proximal and mid segments of LAD [6,8,11-14]. Studies focusing on coronary stenoses by use of coronary flow turbulence and stenotic SPVR have reported high accuracy for detecting stenotic lesions in the LM and LAD, but lower precision for detection of stenoses in the Cx and RCA/RDP [2-4,12]. Incomplete imaging of the individual coronary segments could explain failure of identification of stenotic coronary flow turbulence.

### Limitations of the study

There are several limitations to our study. Use of intravenous ultrasound contrast agent might have improved the results. Because of limited clinical experience in patients with acute coronary syndrome, we chose not to use ultrasound contrast when planning the study. We can not exclude the possibility of overstating the completeness of imaging of the individual coronary artery segments. However, when changing the echocardiographic view we always tried to find overlapping end-portions of the coronary artery compared with the foregoing views, and alternative views were used to fill in any missing portions of the individual coronary artery segment. Moreover, we can not exclude that nearby parallel coursing coronary artery branches occasionally may have been mistaken as the main coronary segment of the dLAD or PDA. In addition, retrograde flow of the distal part of PDA may have been confused with the recurrent branch of the dLAD, which runs around the apex of the heart. Finally, we can not exclude selection bias in our results, since our study cohort only included patients planned for coronary angiography and excluded patients with previous coronary artery bypass surgery, presumed insufficient acoustic windows, significant valvular disease or atrial fibrillation.

## Conclusions

Complete visualisation of the various main coronary artery segments is dependent on both the artery and segment investigated. TTE is a feasible method for complete demonstration of antegrade coronary flow in the LM, the proximal Cx and the different segments of LAD, but less suitable for RCA and mid and distal segments of Cx. Complete visualisation of individual coronary segments might ease the demonstration of coronary stenoses by TTE. Detection of segmental retrograde coronary flow by TTE corresponds well with coronary angiography.

## Competing interests

The authors declare that they have no competing interests.

## Authors' contributions

JV conceived the study, carried out the ultrasound examinations and drafted the manuscript, EH carried out the ultrasound examinations and drafted the manuscript, RW carried out the angiography readings and helped to draft the manuscript, KH carried out the angiography readings, TH participated in the design of the study, performed the statistical analyses and helped to draft the manuscript. All the authors read and approved the final manuscript.
